# Primary gastrointestinal amyloidosis with gastrointestinal hemorrhage and intestinal pseudo-obstruction: a report of a rare case

**DOI:** 10.1007/s12328-018-00929-9

**Published:** 2018-12-20

**Authors:** Yasutoshi Shiratori, Katsuyuki Fukuda, Takashi Ikeya, Koichi Takagi, Kenji Nakamura

**Affiliations:** Division of Gastroenterology, St. Luke’s International Hospital, 9-1 Akashi-cho chuo-ku, Tokyo, 104-8340 Japan

**Keywords:** Amyloidosis, Gastrointestinal amyloidosis, Hemorrhage, Pseudo-obstruction

## Abstract

Amyloidosis is a syndrome involving amyloid protein deposition in various organs, resulting in organ dysfunction. Symptoms of gastrointestinal amyloidosis are usually nonspecific, such as diarrhea and body weight loss. We, here, report a patient who presented to the hospital with simultaneous hematemesis, melena, and intestinal pseudo-obstruction, leading to a diagnosis of primary gastrointestinal amyloidosis based on computed tomography (CT) and endoscopic findings. CT showed diffuse wall thickening from the duodenum to the jejunum, jejunal dilation, and fluid accumulation throughout the gastrointestinal tract. Upper gastrointestinal endoscopy revealed duodenal mucosal edema, jejunal dilation, and small hemorrhages from jejunal mucosal erosion. The definite diagnosis was done based on biopsy results. This report describes the early diagnosis of gastrointestinal amyloidosis based on CT and endoscopy findings.

## Introduction

Amyloidosis is a syndrome that involves amyloid protein deposition in various organs throughout the body, resulting in organ dysfunction [1]. Gastrointestinal amyloidosis includes amyloid A (AA) amyloidosis and amyloid light-chain (AL) amyloidosis [2]. The onset of gastrointestinal amyloidosis usually includes nonspecific symptoms, such as diarrhea and body weight loss; due to the variety of symptoms, diagnosis can be very difficult. Due to the limited number of reports, the association between gastrointestinal amyloidosis and complications, such as gastrointestinal bleeding and intestinal obstruction is not known. It is reported that abdominal computed tomography (CT) in patients with gastrointestinal amyloidosis sometimes show normal bowel findings, and if any abnormal findings are present, gastrointestinal wall thickening and bowel wall dilation will be seen [3].

Here, we report a patient who presented to the hospital with simultaneous hematemesis, melena, and intestinal pseudo-obstruction, leading to a diagnosis of primary gastrointestinal amyloidosis. This report appropriately describes the novel aspects of the study, including the early diagnosis made by CT and endoscopy findings.

## Case report

**Fig. 1:**
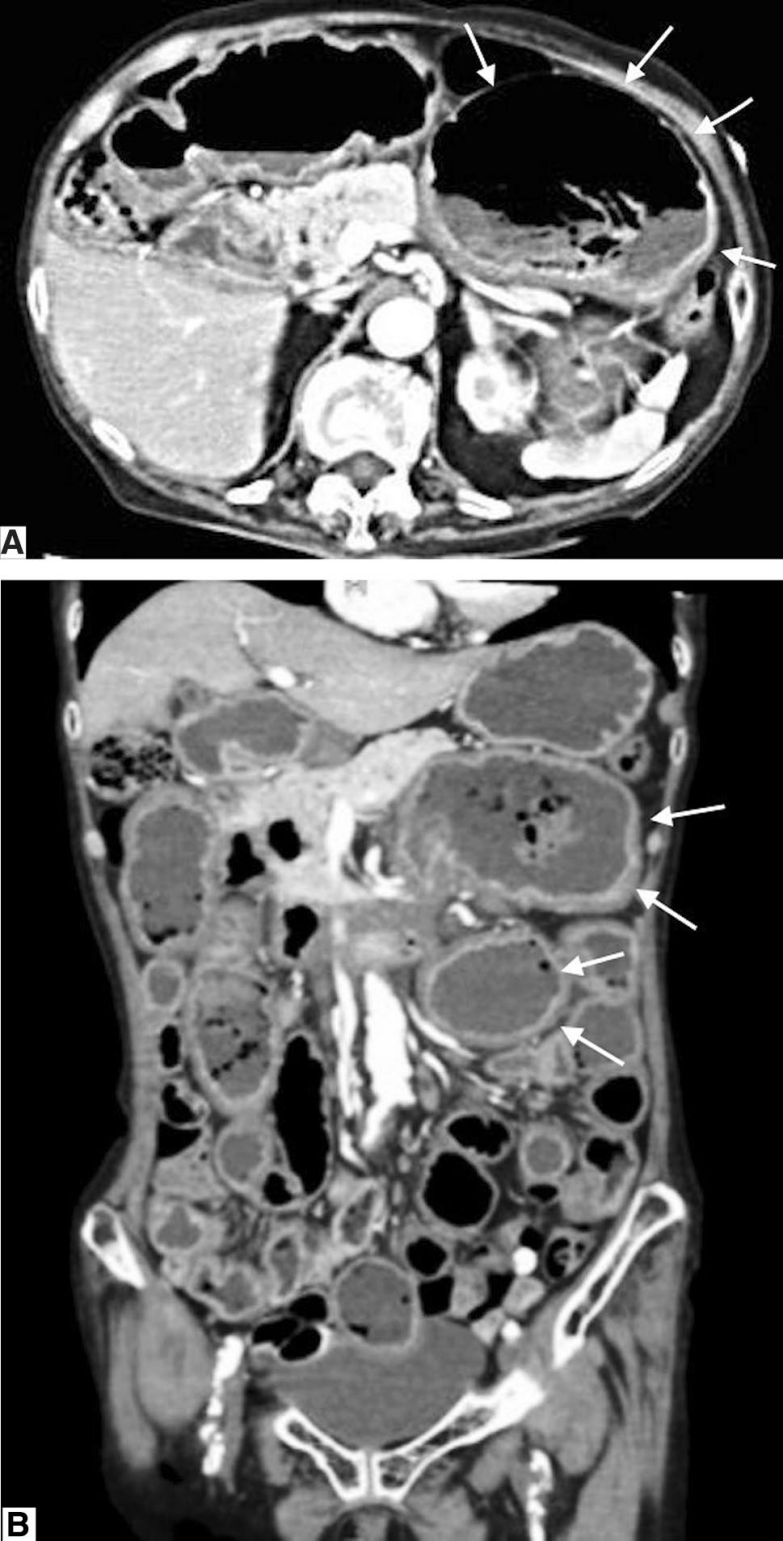
**a** Abdominal computed tomography on presentation. Arrows show marked dilation of the proximal jejunum. **b** Arrows show diffuse intestinal wall thickening and fluid accumulation from the duodenal horizontal portion through the entire jejunum

**Fig. 2:**
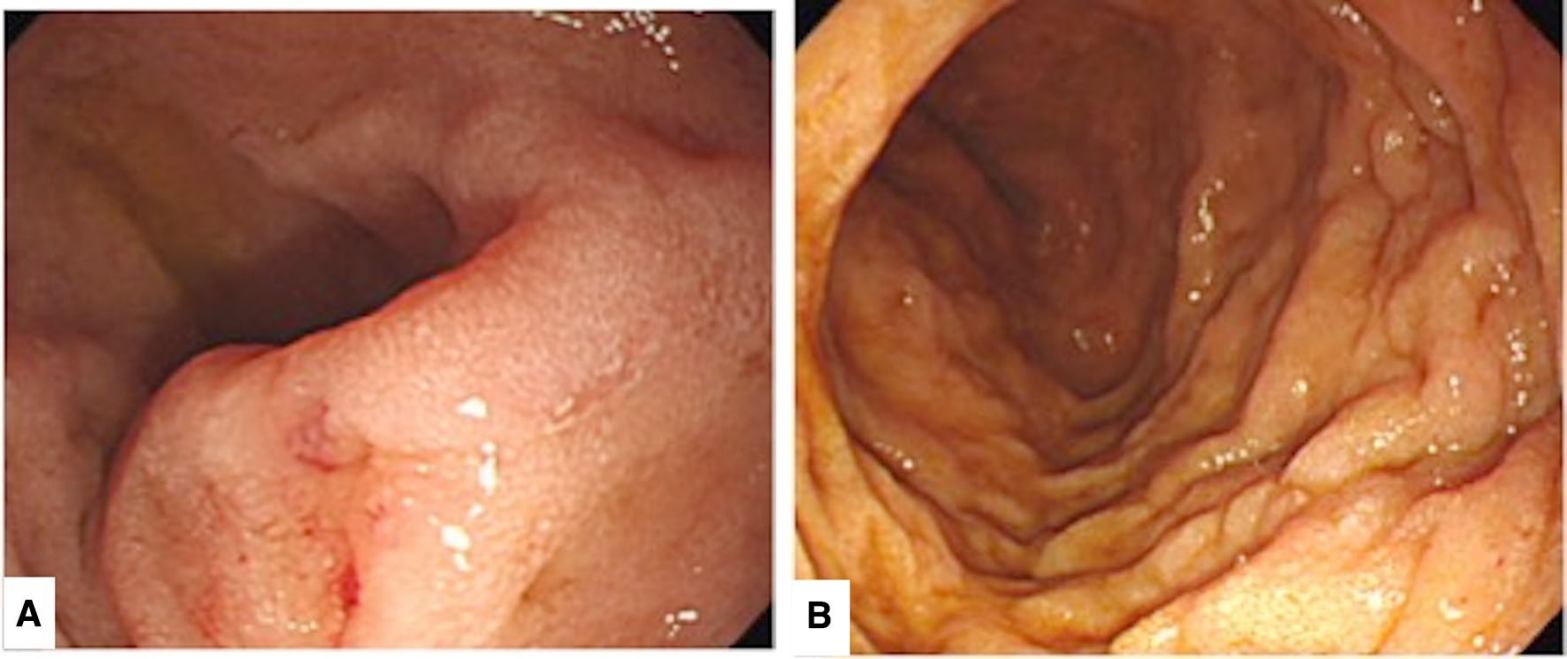
**a** The endoscopic finding of duodenal and jejunum. A submucosal tumor-like protrusion in second portion of duodenum. **b** Showing duodenal mucosal edema and thickening of Kerckring’s fold

**Fig. 3:**
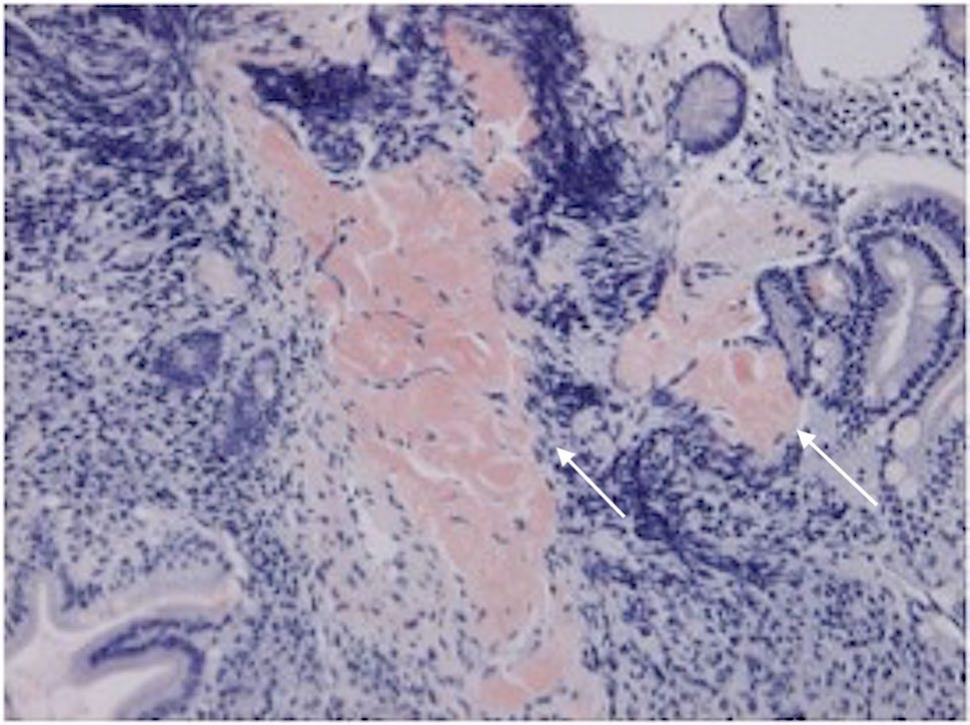
The histological findings showed amyloid deposition was visualized on staining with Congo red. Arrows indicate amyloid proteins that stain red and are clearly distinguished

The patient was an 87-year-old female with a history of hypertension, type 2 diabetes mellitus (no medication, no neurological symptom, just follow-up), angina pectoris (she had medication such as β blocker and nitrate), and prior surgery for appendicitis. In early June 2016, she presented to our hospital emergency department with the primary complaints of hematemesis after eating, repeated vomiting, and melena. She was alert, and her body temperature was 36.7 °C. The blood pressure was 130/68 mmHg, and the regular heart rate was 76 beats/min. Her palpebral conjunctivae were indicative of anemia; there was tenderness in the epigastrium and peristaltic depression. A blood sample was collected (Table 1), and anemia and hypoproteinemia were detected. Abdominal CT showed diffuse intestinal wall thickening and fluid accumulation from the duodenal horizontal portion through the entire jejunum, and marked dilation of the proximal jejunum to a maximum diameter of 7.5 cm (Fig. 1). Although intestinal obstruction-like symptoms were confirmed, no obvious mechanism of obstruction could be identified; thus, a diagnosis of intestinal pseudo-obstruction was made. A clear trigger was not pointed out and a chronic progressive course was suspected. We considered malignant lymphoma, eosinophiric enteritis, SLE enteritis and Crohn disease as differential diseases. Upper gastrointestinal endoscopy was performed in emergency, duodenal mucosal edema, submucosal tumor-like protrusion, and duodenal dilation (Fig. 2) were observed. A biopsy was performed, and samples from the duodenum and jejunum stained with hematoxylin-eosin showed inflammatory cell infiltration, primarily of the plasma cells; amyloid deposition was visualized on staining with Congo red (Fig. 3). Immunostaining for amyloid A was negative, serum amyloid protein was low (5.0 µg/mL), and AL amyloidosis was diagnosed.

**Table 1:** Laboratory test on admission

Hematological value		Biochemical test		Immunology	
WBC	4300 /µL	AST	17 IU/L	IgG	646 mg/dL
RBC	268 × 10^4^/µL	ALT	16 IU/L	IgA	450 mg/dL
Hb	8.3 g/dL	ALP	781 IU/L	IgM	27 mg/dL
Plt	18.5 × 10^4^/µL	LDH	116 IU/L	TSH	1.9 µIU/L
		γ-GTP	5 IU/L	freeT3	2.2 pg/mL
Coagulation test		Na	138 mEq/L	freeT4	1.08 ng/dL
PTINR	0.95	K	4.7 mEq/L	RF	< 6
APTT	23.9 S	Ca	8.1 mg/dL		
		BUN	16.5 mg/dL		
Urine test		Cre	0.38 mg/dL		
U-protein	(−)	T-Bil	1.1 IU/L		
U-glucose	(−)	TP	4.9 g/dL	Tumor marker	
U-OB	(−)	Alb	2.6 g/dL	CEA	0.8 ng/mL
U-BJP	(−)	CRP	0.04 mg/dL	CA19-9	2.9 IU/mL
		Glucose	104 mg/dL		
		HbA1c	4.7%		
		LDL-Cho	55 mg/dL		
		TG	73 mg/dL		

**Fig. 4:**
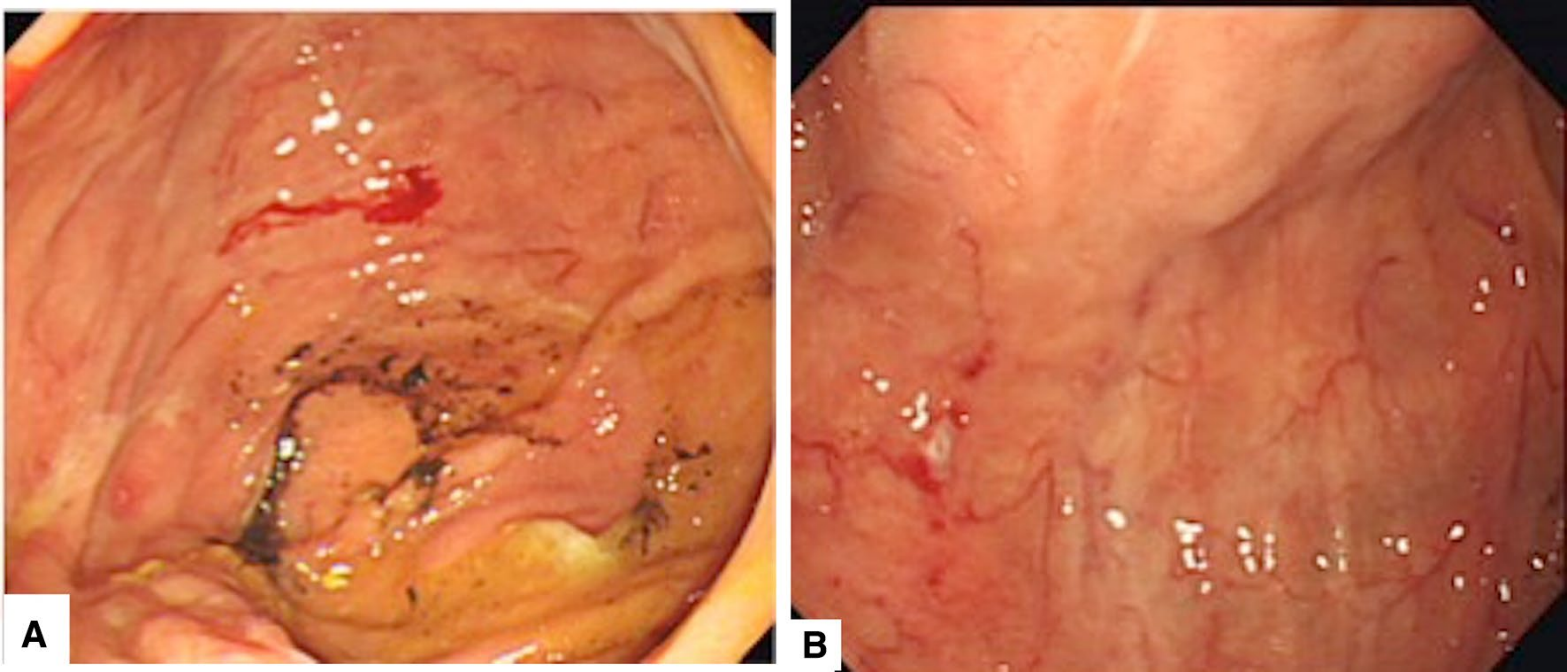
**a** Remarkable jejunal dilation and small hemorrhages from jejunal mucosal erosion. **b** Showing small ulcer in jejunum

We performed lower gastrointestinal endoscopy 2 days later, but no inflammatory findings were observed and no abnormality was shown in biopsy. In addition, we examined small intestine with long scope and confirmed small ulcer and small bleeding in jejunum (Fig. 4). As a cause of melena, we speculated as duodenal erosion, small ulcer in jejunum, and small intestine that could not be observed. It was speculated that continuous mild bleeding caused the decrease the level of Hb. We considered the investing of small intestine.

After hospital admission, the treatment included refraining from intake of food orally, gastrointestinal pressure reduction by insertion of a stomach tube, and administration of proton pump inhibitors, resulting in the resolution of the patient’s symptoms. We administrated her medications that improve peristalsis and enteral feeding product. Following admission, she was discharged on day 5.

After the discharge, we managed the patient by administering intestinal motility improving medications and oral nutrients, and no recurrence of symptoms was observed.

Regarding the informed consent, we got the informed consent prior to inclusion in the study.

## Discussion

There were two important clinical findings with respect to the patient reported here. She presented to the hospital with gastrointestinal amyloidosis, with the primary complaints of hematemesis, melena, and intestinal obstruction and the differential diagnosis of intestinal amyloidosis was predicted on the basis of characteristic CT findings.

First, the usual symptoms of gastrointestinal amyloidosis are body weight loss, diarrhea, constipation and fatigue [4]. In the database search, five cases of patients presenting with the primary complaint of hematemesis were found in the database of the Japan Medical Abstracts Society, and 22 cases were found on PubMed. With respect to intestinal pseudo-obstruction, nine cases were found in the database of the Japan Medical Abstracts Society, and 36 cases were found on PubMed [5, 6]. There are just three cases similar to the case presented here, reporting both, gastrointestinal hemorrhage and intestinal pseudo-obstruction [7–9] (Table 2). When amyloid protein deposition is primarily in the blood vessels, hemorrhage and hematoma formation occur, in addition to protein leakage. In contrast, intestinal peristaltic disorders are considered to develop in cases of amyloid protein deposition extending into the Auerbach’s plexus [10]. The coexistence of both gastrointestinal hemorrhage and pseudo-obstruction of the intestine should alert the clinician to a diagnosis of gastrointestinal amyloidosis.

**Table 2:** Major Case Series; Gastrointestinal amyloidosis patients who have both of gastrointestinel hemorrhage and pseudo-obstruction

No.	Author	Age	Sex	Symptom	Bleeding location	Obstruct location	Type	Complications
1	Iwahashi et al.	73	F	Melena	Intestine	Intestine	AL	MM
2	Lau et al.	60	F	Vomit	Stomach	Duodenum	AL	None
3	Rachelle et al.	70	M	Hematochezia	Intestine	Intestine	AL	MM

*MM* multiple myeloma

Second, on the basis of characteristic CT findings, including diffuse wall thickening from the duodenum to the jejunum, jejunal dilation, and fluid accumulation throughout the gastrointestinal tract, we considered the potential diagnosis of intestinal amyloidosis before endoscopy was performed. Patients with AL amyloidosis, as in the present case, show thickening of the submucosal layer from the muscularis mucosa [11]. In contrast, in AA amyloidosis, the lamina propria is most prominently involved and in many cases, there is little noticeable wall thickening prior to this development [12].

The present patient had no significant underlying disease; thus, the diagnosis of primary AL amyloidosis was made. She had no symptoms related with amyloidosis such as neurologic abnormality, joint ache, anasarca, purple spot and enlarged lymph nodes. Electrophoresis, urinalysis, and cranial CT were performed, but findings suggestive of multiple myeloma were not identified, and rapid alleviation of symptoms was achieved with symptomatic treatment. Clinicians should consider the potential diagnosis of intestinal amyloidosis based on the presenting symptoms and characteristic CT findings.
